# Detection of *Bacillus* Species with Arsenic Resistance and Plant Growth Promoting Efficacy from Agricultural Soils of Nepal

**DOI:** 10.1155/2022/9675041

**Published:** 2022-07-19

**Authors:** Lil Budha Magar, Binod Rayamajhee, Sujan Khadka, Gaurab Karki, Alina Thapa, Muhammad Yasir, Sandeep Thapa, Om Prakash Panta, Suprina Sharma, Pramod Poudel

**Affiliations:** ^1^Department of Microbiology, National College, Tribhuvan University, Kathmandu, Nepal; ^2^School of Optometry and Vision Science, Faculty of Medicine and Health, UNSW, Sydney, Australia; ^3^Department of Infection and Immunology, Kathmandu Research Institute for Biological Sciences (KRIBS), Lalitpur, Nepal; ^4^Department of Microbiology, Birendra Multiple Campus, Tribhuvan University, Bharatpur, Chitwan, Nepal; ^5^Department of Biological Sciences, The University of Toledo, Toledo, Ohio, USA; ^6^Department of Microbiology, Balkumari College, Tribhuvan University, Bharatpur, Chitwan, Nepal; ^7^Kathmandu Center for Genomics and Research Laboratory (KCGRL), Lalitpur, Nepal; ^8^Central Department of Microbiology, Tribhuvan University, Kirtipur, Nepal; ^9^Research Division, University Grants Commission (UGC), Bhaktapur, Nepal; ^10^Central Department of Biotechnology, Tribhuvan University, Kirtipur, Nepal

## Abstract

Arsenic contamination in soil and water is one of the major environmental problems in multiple countries including Nepal imposing a serious threat to the ecosystem and public health. Many soil bacteria can detoxify arsenic, including genus *Bacillus*. With an objective to gauge the plant growth-promoting activities of arsenic-resistant *Bacillus* species, 36 samples (soil, rice, cauliflower, and beans) were collected from the *Terai* region of Nepal. For selective isolation of *Bacillu*s species, each sample was heated at 80°C for 15 min before the inoculation into nutrient agar (NA). Following the standard protocol, arsenic-resistant *Bacillus* species were screened using NA supplemented with 100 ppm sodium arsenate and sodium arsenite. Among 158 randomly selected isolates, only five isolates were able to tolerate sodium arsenite concentration up to 600 ppm. Notably, all five isolates were able to produce indole acetic acid (IAA), a plant hormone, and solubilize phosphate. Based on biochemical analysis and *16S* rRNA gene sequencing, isolates N4-1, RW, KR7-12, Bhw1-4, and BW2-2 were identified as *B. subtilis* subsp. *stercosis, B. flexus, B. licheniformis*, *B. cereus*, and *B. flexus*, respectively. To the best of our knowledge, this is the first study showing the presence of arsenic-resistant *B. flexus* in Nepalese soil with plant growth-promoting traits. Possible utilization of these *Bacillus* strains could facilitate the novel bioremediation pathway to reduce the toxic effect of arsenic from the soil and water in the *Terai* region of Nepal.

## 1. Introduction

Arsenic contamination, because of its high carcinogenic effect, is one of the most seriously perceived global threats to public health [1]. Arsenic (As), a group A carcinogen, is a trace metalloid element present naturally in bedrock and is released from both natural and anthropogenic activities into the water resources contaminating soil and water ecosystems and finally gaining a foothold in the food chain [1–3]. Even though water contamination with arsenic is already perceived as a major global problem, soil contamination with this element should not be overlooked as several studies have reported its health hazards to humans as well as plants [2, 4–7]. Ingestion of high levels of soil arsenic for years can induce cancer of the skin, bladder, and lung as well as neurological and cardiovascular problems [5]. Similarly, soil arsenic adversely affects the physiology, growth, and grain quality of soil crops [2].

According to World Health Organization (WHO), the permissible limit of arsenic in drinking water is 0.01 ppm. However, the national standard for arsenic in most Asian countries is higher and remains at 0.05 ppm due to economic considerations and the unavailability of modern tools to measure the lower arsenic concentrations accurately [8]. Arsenic is widely distributed in our ecosystem contaminating soil and water resources which greatly affect the health of many people worldwide having different extents of toxicity [7, 9, 10]. Arsenic exists in two inorganic phytoavailable forms in soil: arsenate (As(V), H_2_AsO_4_^−^, and HAsO_4_^2−^) and arsenite (As(III) and H_3_AsO^3−^) [1], where As(III) is more toxic which can inhibit various dehydrogenases such as pyruvate, *α*-ketoglutarate, and dihydrofolate. On the other hand, arsenite (AsO_2_^−^ or AsO_3_^− −^) can bind with sulfhydryl groups of proteins and dithiols like glutaredoxin [11]. Plants, as well as humans and animals, are negatively affected by As, which deactivates enzymes and alters metabolic pathways [12]. Some studies have reported that both As(V) and As(III) slow, arrest, or disrupt plant metabolism, impairing reproductive ability and resulting in a loss of yield once taken up by the cells [12, 13].

In the *Terai* region of Nepal, the arsenic problem in tube well water which is used for drinking purpose was reported more than two decades ago; however, little attention was paid to this public health issue (WHO, 2001). The Department of Water Supply and Sewage (DWSS) of Nepal with the technical assistance of WHO has assessed groundwater arsenic in three districts of *Terai*, Jhapa, Morang, and Sunsari, for the first time in 1999 [14], and high arsenic concentration (up to 0.17 mg/L) in other districts of *Terai* (Nawalparasi, Rautahat, Bara, and Bardia) was reported [15, 16]. Similarly, arsenic contamination of fertile soil is a serious problem in Nepal. A study conducted by Dahal et al. has reported an arsenic concentration ranging from 6.1 to 16.7 mg/kg in fertile soil of the *Terai* region where the order of arsenicconcentration in plants was found to be roots > shoots > leaves > edible parts and were observed in different plants (onion leaves, onion bulb, cauliflower, rice, brinjal, and potato) [17]. Consumption of arsenic-contaminated water as well as plants grown in arsenic-contaminated soil can cause several health problems to humans as well as plants [2, 5].

To minimize the higher concentration of arsenic in soil, studies are exploring sustainable and eco-friendly techniques as novel biotreatment. In this regard, the use of microorganisms is receiving much attention due to their diverse applications in bioremediation [8]. A wide range of bacteria are capable of utilizing arsenic compounds as electron donors and electron acceptors or possess arsenic detoxification mechanisms and are collectively named arsenic-resistant bacteria (ARB) [18]. Bacterial strains of genera *Acidithiobacillus*, *Bacillus*, *Deinococcus*, *Desulfitobacterium,* and *Pseudomonas* have already been reported as ARB [19], and these bacteria play a vital role in plant growth by removing metal-induced toxicity and also promote their growth by solubilizing phosphate and by producing various growth-promoting substances such as siderophores, exopolysaccharides (EPS), ammonia, and indole acetic acid (IAA) among others [9].

Nowadays, *Bacillus* species have gained large interest due to its role in arsenic bioremediation, enzyme development, plant growth-promoting (PGP) traits, organic acid production, etc. [20–23]. Since *Bacillus* species are widely present in the environment including arsenic and its form-enriched environment, long-term exposure of arsenic to these could develop arsenic-resistant mechanisms such as arsenite methylation and arsenite oxidation [24]. Therefore, exploitation of *Bacillus* species from such an environment for bioremediation of arsenic is of utmost importance. Chromosomal or plasmid-borne *ars*C genes have been reported in *Bacillus* species [25], and they are capable of removing arsenic from the contaminated environment [26].

Despite the higher concentration of arsenic in water and soil, across the *Terai* region of Nepal, limited studies have been reported to isolate ARB and use them for bioremediation of arsenic-contaminated water and soil. Therefore, this study was designed with an aim to isolate and characterize the *Bacillus* species from soil samples collected from *Terai* districts of Nepal which were resistant to the high concentration of arsenic with an added benefit of plant growth-promoting (PGP) traits.

## 2. Materials and Methods

### 2.1. Site Description and Sample Collection

Thirty-two samples (*n* = 36) comprising 18 topsoils, 9 water, 5 rice grains, 3 beans, and 1 cauliflower were collected from eleven districts of the *Terai* region, Nawalparasi, Mahottari, Sunsari, Chitwan, Bara, Rautahat, Rupandehi, Jhapa, Kailali, Bhairahawa, and Sarlahi, between February and March 2019. Samples were collected in a separate sterile Ziploc bag and transported to the laboratory of National College, Kathmandu, where samples were stored at 4°C until further analysis.

### 2.2. Processing of Soil Samples

Samples were processed according to the protocols followed by Khadka et al. [22] and Sapkota et al. [23]. Ten grams (g) of each sample was transferred separately in 90 mL of 0.85% (w/v) sterile normal saline and heated in a water bath at 80°C for 15 min with constant stirring. Tenfold serial dilution was prepared up to 10^−6^ for each sample, and then, 0.1 mL of an aliquot from the dilutions of 10^−2^, 10^−4^, and 10^−6^ were aseptically inoculated in sterile nutrient agar (NA). Plates were incubated at 37°C for 48 h and observed for typically *Bacillus*-like colonies [23, 27].

### 2.3. Screening of Arsenic-Resistant *Bacillus* Species and Silver Nitrate Test

Morphological and physiological characterization of isolated bacterial colonies were carried out as described elsewhere [28]. In brief, *Bacillus*-like colonies were randomly selected and were subcultured in freshly prepared NA plates supplemented with different concentrations of sodium arsenite (100 to 600 ppm) (HiMedia, Mumbai, India) and incubated at 37°C for 48 h. After incubation, those colonies showing different degrees of resistance to arsenite were selected and subcultured by a single-line streak-plate method on fresh NA plates supplemented with sodium arsenite and incubated at 37°C for 48 h. The transformation ability (oxidation or reduction) of arsenic by the isolates was screened using the AgNO_3_ method as performed by Tiwari et al. [29] with slight modifications. After incubation, agar plates were flooded with the solution of 0.1 M AgNO_3_ (HiMedia, Mumbai, India). A brownish precipitate showed the presence of arsenate in the medium (arsenite-oxidizing bacteria), while the presence of arsenite was detected by a bright yellow precipitate (arsenate-reducing bacteria) [30].

### 2.4. Biochemical Characterization

Different biochemical tests (oxidase, catalase, indole, gelatinase, urease, and amylase), salt tolerance, carbohydrate fermentation, amino acid utilization, H_2_S production ability, citrate utilization, and nitrate reduction ability of isolated *Bacillus* species were examined. The biochemical properties of the isolates were assessed following the guidelines of Bergey's Manual of Systematic Bacteriology, Volume 3 [28].

### 2.5. Plant Growth-Promoting (PGP) Traits of Isolated *Bacillus* Species

Different PGP traits of isolated *Bacillus* species such as phosphate solubilization and production of indole acetic acid (IAA) and ammonia were examined in *in vitro* conditions. Phosphate-solubilizing property was estimated following the method of Fiske and Subbarow on Pikovskayas agar plates (HiMedia, Mumbai, India). In brief, *Bacillu*s colony was streaked on solid Pikovskayas agar and it was incubated at 28°C for 7 days. A zone of hydrolysis around bacterial colonies suggests phosphate solubilization [31]. A qualitative analysis of IAA production by the isolates was evaluated as described by Loper et al. [32] at different concentrations of tryptophan (0.06 g/L, 0.12 g/L, 0.18 g/L, and 0.25 g/L). To determine the amount of IAA produced by each isolate, a colorimetric technique was performed with Van Urk Salkowski's reagent. Isolates were grown in yeast malt dextrose broth (YMDB) (HiMedia, Mumbai, India) and incubated at 28°C for 4 days. After incubation, the broth was centrifuged at 3,000 rpm for 30 min; then, the supernatant was collected and mixed with two drops of orthophosphoric acid and 4 mL of Salkowski's reagent (2% 0.5 FeCl_3_ in 35% HClO_4_ solution) and was kept in a dark place. The optical density (OD) was recorded at 570 nm after 30 min and 120 min [33]. Ammonia production by the isolates was examined by inoculating the fresh culture in peptone water and incubated for 48 h at 28°C. Production of yellow to brown precipitate after the addition of 0.5 mL Nessler's reagent on an incubated tube indicated ammonia production [34].

### 2.6. Antibiotic Sensitivity

The antibiotic sensitivity test (AST) of the arsenic-resistant *Bacillus* species was determined by the standard disc-agar diffusion (Kirby–Bauer) method [35, 36]. Antibiotic-impregnated discs (6 mm diameter, HiMedia, India, containing ampicillin (10 *µ*g), bacitracin (10 *µ*g), chloramphenicol (30 *µ*g), and erythromycin (10 *µ*g)) were placed on Mueller–Hinton agar (MHA) plates (Oxoid, Hampshire, UK) previously swabbed with respective arsenic-tolerant bacterial suspension of 0.5 McFarland standard prepared in sterile water. Plates were incubated at 37°C for 24 h. The diameter of the zone of inhibition (ZoI) was measured, and the interpretation was made as per the ZoI size interpretation value. To adjust the quality control of AST, *E. coli* ATCC 25922 and *Staphylococcus aureus* ATCC 25923 were used.

### 2.7. The Effect of Arsenic on Bacterial Growth

The growth of ARB strains was determined using nutrient broth (NB). From an overnight pure culture, 1% inoculum was added to 50 mL of the NB medium supplemented with 200 ppm, 400 ppm, 600 ppm, 800 ppm, 1,000 ppm, and 1,300 ppm sodium arsenite and incubated at 37°C in a shaker (120 rpm) for 72 h. The growth of the isolate was monitored by measuring optical density at 600 nm using a spectrophotometer [9].

### 2.8. Molecular Identification of Potent Arsenic-Resistant *Bacillus* Species

Genomic DNA of isolated *Bacillus* species was extracted by phenol-chloroform assay, and DNA amplification of the *16S* rRNA gene was performed by the primer sets: 8F (5′-AGAGTTTGATCCCTCAG-3′) and 1492R (5′-GGTTACCTTGTTACGACTT-3′) [22, 34, 37, 38]. PCR amplification conditions were as follows: 30 cycles of denaturation at 98°C for 10 seconds and annealing at 55°C for 5 seconds with final elongation at 72°C for 1 min. PCR products were purified using QIAquick PCR purification kit according to the manufacturer's instructions. Sequence homology was compared with *16S* rRNA gene sequences available in the DDBJ/EMBL/GenBank DNA database using the FASTA algorithm (https://www.ddbj.nig.ac.jp/), and all reference sequences were obtained through the Ribosomal Database Project II (https://rdp.cme.msu.edu/). Sequences were aligned using CLUSTAL W ver.2.01 (https://clustalw.ddbj.nig.ac.jp/), and the phylogenetic tree was constructed using MEGA ver.7 by the neighbour-joining method with bootstrap values calculated from 1,000 replications [39].

### 2.9. Data Analysis

The data analysis and plot constructions were performed by using the R programming statistical analysis tool (version 1.2.5033) with ggplot2 (grammar of graphics) (version 3.3.2) (https://cran.r-project.org/). All experiments were conducted in triplicate, and mean and standard deviation (SD) and were measured and presented as mean ± SD.

## 3. Results

### 3.1. Isolation and Identification of Arsenic-Resistant Bacteria (ARB)

**Figure 1:**
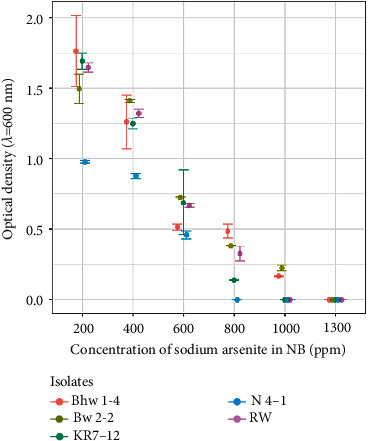
Growth of isolates in the arsenite-containing medium.

One hundred fifty-eight isolates were obtained from 36 processed samples. The sample description and the total number of colonies randomly selected in this study are depicted in Table 1. Screened colonies were flat, irregular, moist, and slightly convex. Of 158 isolates, only five isolates, Bhw 1-4, RW, N 4-1, KR 7-12, and BW 2-2, were able to grow on nutrient broth containing sodium arsenite concentration of 600 ppm. Two isolates BW2-2 and Bhw1-4 tolerated up to 1000 ppm sodium arsenite (Figure 1).

### 3.2. Arsenic-Oxidizing Ability of the Isolates

**Figure 2:**
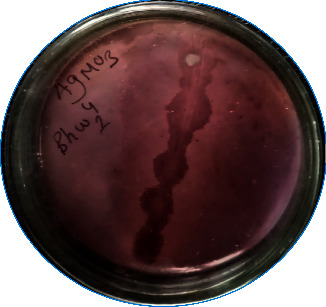
Silver nitrate test of isolate Bhw1-4 after incubation for 3 days at 37°C in NA containing 600 ppm sodium arsenite.

All five arsenic-resistant isolates (N4-1, RW, KR7-12, Bhw1-4, and BW 2-2) have oxidized As(III) to As(V) in the arsenite-containing medium. The brown precipitate was observed after adding silver nitrate solution in NA after 3 days of incubation at 37°C (Figure 2).

### 3.3. Plant Growth-Promoting Traits

**Figure 3:**
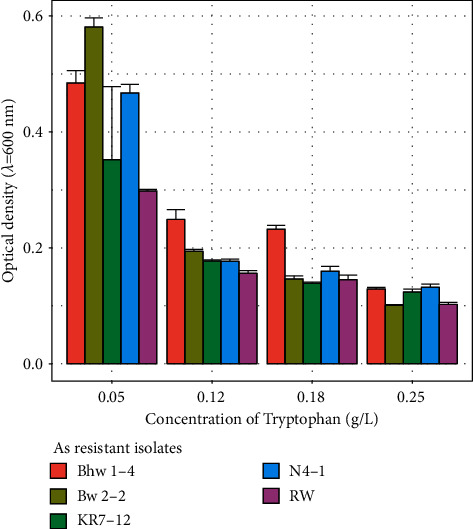
Growth of isolates at different concentrations of tryptophan.

Two isolates N4-1 and KR7-12 produced ammonia and solubilized phosphate in Pikovskayas agar, while Bhw1-4 isolate only utilized phosphate. IAA-producing isolates are summarized in Figure 3. The maximum absorbance of 0.581 ± 0.016 was observed in isolate BW2-2, and minimum absorbance of 0.298 ± 0.003 was seen in isolate RW in culture broth containing a tryptophan concentration of 0.05 g/L. Growth was decreased with an increase in tryptophan concentration.

### 3.4. Hydrolysis and Antibiotic Sensitivity Tests

**Figure 4:**
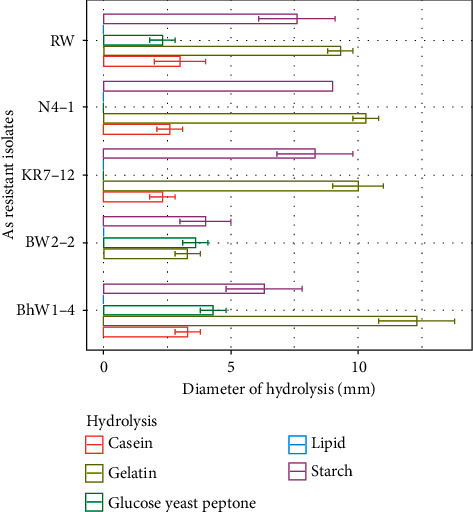
Hydrolysis test of the isolates.

All five isolates hydrolyzed starch and gelatin. Similarly, all isolates also hydrolyzed casein except BW 2-2, but none of the isolates hydrolyzed lipid (Figure 4). The antibiotic susceptibility test (AST) showed that isolates were sensitive to chloramphenicol, erythromycin, and ampicillin (Table 2).

### 3.5. Sugar Assimilation Pattern

Isolates KR7-12, BW2-2, RW, Bhw1-4, and N4-1 were able to ferment sugars such as glucose, fructose, lactose, sucrose, galactose, mannose, mannitol, maltose, and xylose (Table 3). Based on the sugar assimilation pattern, isolates were phenotypically confirmed as *Bacillus* species.

### 3.6. Growth Pattern of the Isolates under Different NaCl Concentrations and pH Values

**Figure 5:**
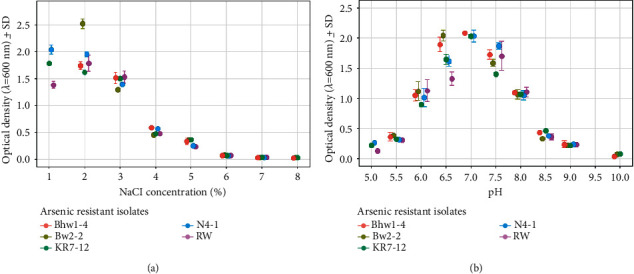
Growth of arsenic-resistant isolates under different NaCl concentrations (a) and pH values (b).

The optimum sodium chloride (NaCl) concentration for the growth of arsenic-resistant isolates KR7-12, BW2-2, RW, Bhw1-4, and N4-1 varied from 1 to 2%. Similarly, isolates showed growth at pH range 5–9 with optimum being at pH 7 (Figure 5).

### 3.7. Molecular Characterization of Isolates

**Figure 6:**
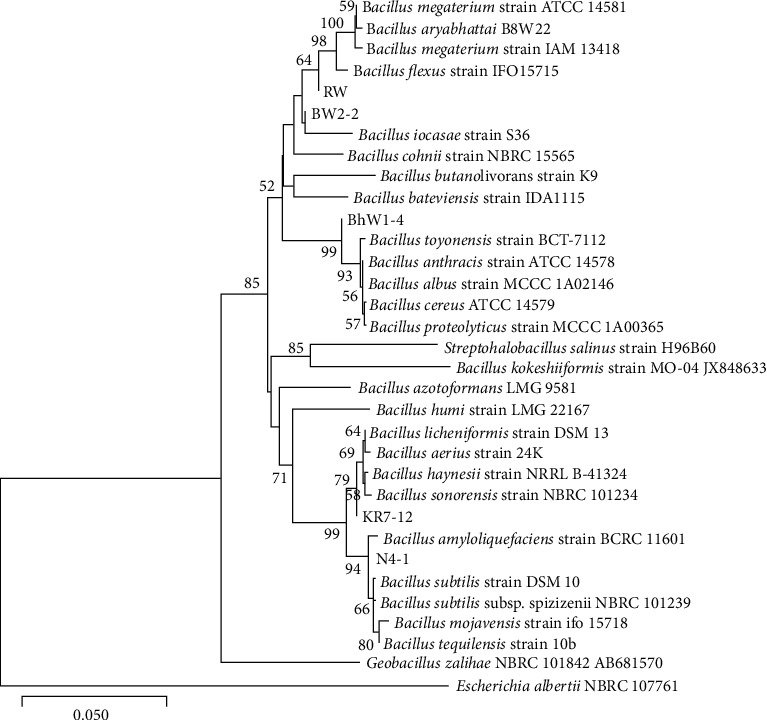
Neighbor-joining tree based on *16S* rRNA gene sequences, showing the position of isolates RW, BW2-2, Bhw1-4, KR7-12, and N4-1, and their closely related reference strains inclusive of other *Bacillus* species. Bootstrap values (expressed as percentages of 1000 replications) above 50% are shown. Bar 0.01 substitutions per nucleotide position. *Escherichia albertii* NBRC 107761 is used as an outgroup.

Based on a comparative analysis of the *16S* rRNA with those available in the database, it was observed that the isolate Bhw1-4 showed 99.4% similarity with *Bacillus cereus*. Isolates RW and BW2-2 showed 99.2% and 99.4% similarity with *B. flexus*, respectively, N4-1 showed 99.5% similarity with *B. subtilis* subsp. stericosis, and isolate KR7-12 showed the highest (98.8%) similarity with *B. licheniformis*. The neighbour-joining tree based on *16S* rRNA gene sequences, showing the position of isolates and the closely related species of the genus *Bacillus*, is depicted in Figure 6.

## 4. Discussion

Isolation and characterization of ARB is a primary step to determine their abilities in the bioremediation of heavy metals. In this study, arsenic-resistant *Bacillus* species were identified and characterized from 36 different samples collected from the *Terai* region of Nepal. A high concentration of arsenic in the drinking water of the *Terai* region has been a long-standing public health issue of Nepal [40]. The soil profile of processed samples in this study reveals a slightly acidic pH (5-6); while soil microbes exhibit tolerance to a wide range of pH (3–13) [41]. Among 158 isolates of *Bacillus* species, five isolates N 4-1, RW, KR7-12, Bhw 4-1, and BW2-2 were found to be arsenic tolerant in culture media and could grow on NB containing a sodium arsenite concentration of >600 ppm. This study indicates the abundance of arsenic-resistant *Bacillus* species in agricultural soil, water, and rice of Nepal, which is in accordance with a study conducted by Shakya et al. [36]. In addition, studies conducted by Selvi et al. [42], Majumder et al. [43], and Bachate et al. [44] have also reported the isolation of ARB species from the soil of different parts of the world. A study by Joshi et al. [45] examined higher ARB strains from industrial effluents in India. Evidence from several previous studies [36, 42–45] reported the diversity of the *Bacillus* species from different environmental niches with varying capabilities of exhibiting resistance to arsenic, motivating the search for more potent *Bacillus* species in Nepal. As a result, the attempt to study the ARB as well as their plant growth-promoting activity in Nepal has reported ARB from various samples such as soil, water, rice, bean, and cauliflower possessing some remarkable properties such as high arsenic tolerance and the ability to transform toxic As(V) to less toxic As(III). These characteristics, along with ARB's plant growth-promoting capabilities, could play a significant role in reducing arsenic from fertile soil with increased plant growth in Nepal. However, to accomplish these goals, further in-depth studies must be done which could require a high level of scientific, political, and systematic efforts.

In addition, isolated species also exhibited plant growth activity directly by producing IAA and solubilizing phosphate and indirectly by removing toxic arsenic from the soil. In the context of arsenic contamination as a major environmental health management issue of Nepal, especially in the *Terai* region, isolation and molecular characterization of arsenic-resistant *Bacillus* species could facilitate bioremediation to reduce toxic heavy metals such as arsenic in the drinking water and soil of the region and beyond.

*Bacillus* strains isolated in this study showed a higher degree (>600 ppm) of resistance to sodium arsenite. The presence of arsenic in environmental resources enriches the ARB [19]. But in this study, soil, water, rice, bean, and cauliflower samples were collected from different agricultural fields and measurement of the samples' arsenic content was not performed. Bacterial species isolated from an arsenic-free environment might possess some mechanism to tolerate higher arsenic concentration as reported by Salam et al. [46]. Bachate et al. [44] have analyzed ARB to determine their potential role for the bioremediation of arsenic and have reported that the isolates were highly resistant to arsenic (manyfold higher compared to the arsenic content of the soil). In another study conducted by Poudel et al. [34], a highly arsenic-resistant strain of *Bacillus* species has been reported which can tolerate up to 1,000 ppm and 15,000 ppm of sodium arsenite and sodium arsenate, respectively. The reason why these strains tolerate higher concentration of arsenite and arsenate is still not clear. Bachate et al. [44] have mentioned that high arsenic resistance in some *Bacillus* species may be due to the presence of multiple sets of *ars* operon in their chromosome. The *aoxB* gene in *ars* operon has been used as a genetic marker for As(III) oxidation which is prevalent among the members of the genus *Bacillus* [43]. ARB can oxidize As(III) to As(V) which represents a potential detoxification mechanism as it generates less toxic and less mobile forms of arsenic. This detoxification mechanism has a significant application in bioremediation [47]. In this study, a microplate screening assay was used to assess the arsenite transforming ability of the ARB isolates. All five ARB isolates were screened positive for the As(III) oxidation reaction in the AgNO_3_ screening test. Two of the isolates N4-1 and KR7-12 exhibited both the ability to produce ammonia as well as solubilizing phosphate in Pikovskayas agar, while the other isolate, Bhw1-4, solubilized phosphate only. The ability of soil bacteria to promote plant growth especially in metal-contaminated soil makes the preferred choice for microbial-assisted phytoremediation such as rhizospheric bacteria promoting plant growth by their ability to solubilize phosphate [48]. ARB species with PGP traits have been reported in several studies in which bacteria have shown a positive role in plant growth [49]. Bachate et al. [50] have reported ARB species with important PGP traits that have a direct or indirect influence on plant growth.

All *Bacillus* isolates produced IAA (C_10_H_9_NO_2_) while the production was highest at 0.05% tryptophan in culture. With an increase in tryptophan concentration, the accumulation of IAA declined for all the isolates. On the other hand, in a study reported by Ahmad et al. [51], IAA production was increased in a higher concentration of tryptophan in the culture medium when cocultured with *Pseudomonas* and *Azotobacter* species. Poudel et al. [34] have mentioned this difference might be due to variation in the type of microbes and their sensitivities against tested compounds. All the isolates of this study were able to ferment sugars, such as glucose, fructose, lactose, sucrose, galactose, mannose, mannitol, maltose, and xylose. Referring to the sugar assimilation pattern mentioned in Bergey's manual of determinative bacteriology (1957), the test isolates could be *B. subtilis, B. licheniformis, B. pumilus, B. brevis,* or *Geobacillus stearothermophilus* [28]. Isolates Bhw1-4, KR7-12, and RW were resistant to ampicillin (10 mg), while Rajkumar et al. [48] have reported *Bacillus* species resistant to a wide range of antibiotics including penicillin, ampicillin, kanamycin, and streptomycin indicating the high degree of antibiotic resistance might be associated with heavy metal tolerance. The pH has a crucial role in the growth and metal accumulation properties of the *Bacillus* species [8]. The optimum pH for growth was found to be 7.0 where all the isolates tolerated a pH of range 5–9. Similarly, all the isolates had high salt tolerance where the highest growth was observed at 2% NaCl. The findings of this study are in agreement with the typical characteristics of *Bacillus* species [21].

Phylogenetic study based on *16S* rRNA gene sequence indicated that isolate N4-1 was closely related to *B. subtilis* subsp. *stercosis* with 99.55% similarity, while isolate KR7-12 showed 98.8% similarity with *B. licheniformis*, BhW1-4 showed 99.43% similarity with *B. cereus,* and RW and BW2-2 showed 99% similarity with *B. flexus.*

## 5. Conclusions

*Bacillus* species isolated from the soil, water, rice, bean, and cauliflower possess some interesting properties such as high arsenic tolerance and transform toxic As(V) to less toxic As(III) which could play a crucial role in the reduction of arsenic from the fertile soil of *Terai* region in Nepal. In addition, isolated species also exhibited plant growth activity directly by producing IAA and solubilizing phosphate and indirectly by removing toxic arsenic from the soil. In the context of arsenic contamination as a major environmental health management issue of Nepal especially in the *Terai* region, isolation and molecular characterization of arsenic-resistant *Bacillus* species could facilitate bioremediation to reduce toxic heavy metals such as arsenic in the drinking water and soil of the region and beyond.

## Figures and Tables

**Table 1:** Sample description and the total number of isolates.

Location	Sample code	Sample type^*∗*^	Isolated strain (*n*)

Nawalparasi	NR, NM, N, NG, NW	Topsoil (3), water, rice	31
Chitwan	CM, CB, CR, CW, BT	Topsoil (2), water, bean, rice	28
Rupandehi	ButW, ButM, ButB, ButR, BtlW, But	Topsoil (2), water (2), rice, bean	24
Mahottari	MM, MW, MC, MBM	Topsoil (2), water, cauliflower	14
Bara	BM, BB, BW	Topsoil, water, bean	11
Rautahat	RM, RW	Topsoil, water	8
Jhapa	JM, JD	Topsoil (2)	9
Kailali	KM, KW, KR	Topsoil, water, rice	12
Bhairahawa	Bhw, BhwM	Topsoil (2)	10
Sunsari	IM, IW	Topsoil, water	6
Sarlahi	S, SR	Topsoil, rice	5
Total isolates			**158**

^*∗*^pH of soil ranged from 5.9 to 6.3.

**Table 2:** Antibiotic sensitivity test.

Antibiotics	Zone of inhibition (ZoI) (mm)				
	Bhw1-4	KR7-12	RW	BW2-2	N4-1

Ampicillin	11 ± 0.0	8.5 ± 0.7	13.5 ± 0.7	5.5 ± 0.7	13.5 ± 0.7
Chloramphenicol	12.5 ± 0.7	11.5 ± 0.7	22 ± 0.0	22 ± 1.4	23 ± 0.0
Bacitracin	11 ± 1.4	10.5 ± 0.7	24 ± 0.0	14 ± 0.0	15 ± 0.0
Erythromycin	24.5 ± 0.7	20 ± 1.4	23.5 ± 0.7	23 ± 0.0	7.5 ± 0.7

**Table 3:** Sugar assimilation pattern of isolates.

Sugar/substrate hydrolysis	Isolates				
	KR7-12	BW2-2	RW	Bhw1-4	N4-1
Glucose	**+**	**+**	**+**	**+**	**+**
Fructose	**+**	**+**	**+**	**+**	**+**
Lactose	**+**	**+**	**+**	**+**	**+**
Sucrose	**+**	**+**	**+**	**+**	**+**
Galactose	**+**	**+**	**+**	**+**	**+**
Mannose	**+**	**+**	**+**	**+**	**+**
Mannitol	**+**	**+**	**+**	**+**	**+**
Maltose	**+**	**+**	**+**	**+**	**+**
Xylose	**+**	**+**	**+**	**+**	**+**

Note: “+” = positive.

## Data Availability

The datasets used and analyzed during this study are available in excel sheets which can be obtained from the corresponding author on reasonable request. The assigned DDBJ accession number of the isolates ranged from LC512758 to LC512763. The available link is as follows: https://getentry.ddbj.nig.ac.jp/getentry/na/LC512758/?filetype=htmL.

## Abbreviations

As: Arsenic
DDBJ: DNA Data Bank of Japan
DWSS: Department of water supply and sewage
EPS: Exopolysaccharide
IAA: Indole acetic acid
MHA: Muller–Hinton agar
NA: Nutrient agar
NaCl: Sodium chloride
NB: Nutrient broth
OD: Optical density
PCR: Polymerase chain reaction
PGP: Plant growth promoter
rRNA: Ribosomal ribonucleic acid
WHO: World Health Organization
YMDB: Yeast malt dextrose broth
ZoI: Zone of inhibition.
